# Evaluation of the Prognostic Ability of the Antithrombin/Creatinine Ratio in Critically Ill Patients: Comparison With Sequential Organ Failure Assessment (SOFA) Score and Novel Antithrombin-modified SOFA Score

**DOI:** 10.7759/cureus.100454

**Published:** 2025-12-30

**Authors:** Kenya Yarimizu, Ryuto Yokoyama, Hiroaki Toyama, Kaneyuki Kawamae

**Affiliations:** 1 Department of Anesthesiology, Yamagata University Faculty of Medicine, Yamagata, JPN; 2 Department of Emergency and Critical Care Medicine, Yamagata University Faculty of Medicine, Yamagata, JPN; 3 Department of Anesthesiology, Ohta Nishinouchi Hospital, Koriyama, JPN

**Keywords:** antithrombin deficiency, coagulation parameter, critical care and hospital medicine, life prognosis, sofa score

## Abstract

Purpose: The objective of the study was to evaluate the prognostic ability of the antithrombin/creatinine (AT/Cre) ratio in critically ill patients and compare it with the Sequential Organ Failure Assessment Score (SOFA) score.

Methods: This retrospective cohort study was conducted at two hospitals and included patients admitted to the ICUs between January 2018 and December 2019 who had their AT activity measured within 24 hours of ICU admission. The primary endpoint was mortality at discharge. Statistical analyses included receiver operating characteristic (ROC) curve analysis, K-means clustering, and the Cox proportional hazards model.

Results: The study included 267 patients, of whom 173 (64.8%) were male. The median age was 69 years. The AT/Cre ratio demonstrated prognostic accuracy of mortality at discharge (AUC: 0.71, cut-off: 53.6) comparable to the SOFA score (AUC: 0.75, cut-off: 8.0). A lower AT/Cre ratio was significantly associated with increased mortality risk in multivariate analyses (hazard ratio (HR): 0.98, 95%CI: 0.98-0.99). When patients were categorized into four clusters, AT/Cre values differed significantly between clusters (p <0.0001), with higher values in clusters with a favorable prognosis.

Conclusion: The AT/Cre ratio showed prognostic performance comparable to SOFA, although its incremental value beyond established scores was not evaluated in this study. It is simple to calculate and may be valuable for early risk stratification and treatment decisions.

## Introduction

Accurate prognostic assessment in critical care environments remains fundamental for optimizing therapeutic approaches and resource allocation. Timely and precise outcome prediction, particularly within intensive care settings, facilitates prompt clinical interventions. Although established severity assessment tools such as the Acute Physiology and Chronic Health Evaluation (APACHE) II have gained widespread acceptance, their implementation requires numerous variables and considerable computation time [1,2]. Current trends emphasize developing streamlined prognostic approaches utilizing single biomarkers or limited parameter sets [3-8].

Hemostatic and fibrinolytic system dysfunction represents a cornerstone in critical illness pathophysiology, suggesting that related biomarkers may offer prognostic value [9-12]. Antithrombin (AT), a primary endogenous coagulation inhibitor, becomes depleted during severe infections, septic conditions, and multi-organ failure, correlating with adverse outcomes [13-18]. The Sequential Organ Failure Assessment (SOFA) score incorporates platelet counts as a coagulation indicator, though this may inadequately reflect increased vascular permeability and broader endothelial injury [19,20]. Elevated serum creatinine (Cre), indicating renal impairment, frequently complicates critical illness and is an established adverse prognostic marker.

AT depletion reflects coagulation and endothelial dysfunction, whereas elevated creatinine indicates renal impairment, two pathophysiological disturbances that independently contribute to poor outcomes in critical illness. Importantly, renal impairment can alter AT metabolism and distribution, meaning isolated AT levels may underestimate true coagulation dysfunction in patients with reduced renal function. Previous investigations further suggest that concurrent abnormalities in these pathways may synergistically worsen prognosis. Therefore, integrating AT and creatinine values into a single ratio may offer a simplified yet comprehensive representation of global physiological derangement. The AT/Cre ratio normalizes AT activity against renal function and may better reflect the interaction between coagulation abnormalities and renal dysfunction than either parameter alone. Prior studies demonstrating disproportionately higher mortality in patients with combined coagulation and renal impairment support the rationale for such a composite marker [21].

In parallel with biomarker-based approaches, incorporating AT activity into existing multivariable prognostic tools may enhance their ability to characterize endothelial and coagulation abnormalities. The AT-modified SOFA (ASOFA) score was developed to explore this concept by the authors, although its comparative prognostic utility relative to conventional SOFA scoring remains inadequately defined.

Given these considerations, the present study aims to evaluate the prognostic ability of the AT/Cre ratio for in-hospital mortality in critically ill patients and to compare its performance with the SOFA score. Additionally, we developed the ASOFA score to examine whether integrating AT activity into the SOFA framework enhances prognostic discrimination. By presenting these objectives independently, the study provides an unbiased comparison between a simplified biomarker-derived index and a modified multivariable scoring approach, without presupposing superiority of either method. This investigation further explores the clinical contexts in which these measures may be most informative, particularly among patients exhibiting concurrent coagulation disturbances and renal dysfunction.

## Materials and methods

Materials and methods

This retrospective cohort study was conducted at Yamagata University Hospital in Yamagata, Japan, and Nihonkai General Hospital in Sakata, Japan, incorporating extended monitoring from ICU admission through hospital discharge. Institutional ethics committees from both hospitals approved the study protocol (approval numbers: Yamagata University Hospital 2020-312; Nihonkai General Hospital 2020-29), and all procedures were conducted in accordance with the Declaration of Helsinki.

The study was registered with the University Hospital Medical Information Network (UMIN) Clinical Trials Registry (registration number: R000049113, dated February 1, 2021; https://center6.umin.ac.jp/cgi-bin/ctr/ctr_view_reg.cgi?recptno=R000049113). Informed consent requirements were waived using an opt-out methodology. The primary investigator assumes responsibility for study data integrity and analytical accuracy.

Study population

All patients admitted to participating hospital ICUs between January 2018 and December 2019 were considered for the study. The primary inclusion requirement was AT activity measurement within 24 hours of ICU admission. To ensure comparability of AT measurements across centers, both hospitals followed the same measurement window, obtaining the first AT activity sample as soon as possible after ICU arrival and always within 24 hours of admission. No center-specific differences in sampling policy or timing existed. Exclusion criteria comprised: (i) mortality within 24 hours of ICU admission, (ii) age below 18 years, (iii) AT preparation use before measurement, (iv) pregnancy, (v) congenital AT deficiency, and (vi) HIV infection or acquired immunodeficiency syndrome. No upper age restrictions were applied.

Laboratory assessments

AT activity measurement utilized ACL TOP 750 CTS (Instrumentation Laboratory Company, Bedford, Massachusetts, United States) or CP3000 (Sekisui Medical Company, Tokyo, Japan) analyzers. Manufacturer-specified normal AT activity ranges were 83-128% for ACL TOP 750 CTS and 80-130% for CP3000. Identical instruments measured other coagulation parameters, including fibrin degradation products. Arterial blood was preferred when arterial access was available; otherwise, venous samples were obtained. Blood collection occurred in 3.2% sodium citrate tubes, with plasma extraction through centrifugation at 1500 rpm for 15 minutes. Measurements proceeded promptly following collection, with specimen processing at ambient temperature according to Clinical and Laboratory Standards Institute guidelines.

Outcomes

The primary endpoint was hospital discharge mortality. Secondary endpoints included seven-day and 28-day mortality. This study primarily followed the Transparent Reporting of a multivariable prediction model for Individual Prognosis Or Diagnosis (TRIPOD) reporting guideline [22] for prognostic model evaluation. STandards for Accurate Reporting of Diagnostic accuracy studies (STARD) items [23] relevant to prognostic accuracy reporting were applied where appropriate; however, this study was not a diagnostic accuracy study with a defined reference standard.

ASOFA scoring and evaluation

ASOFA score calculation incorporated AT activity into SOFA scoring. AT activity was stratified into five categories based on mortality-associated thresholds identified in clinical studies. These thresholds were literature-informed but applied in an exploratory manner and were not prespecified for this cohort.: >80% (normal, 0 points) as established by Gazzaruso et al. [24]; 65-79.9% (mild deficiency, 1 point) supported by the 70% threshold validated in sepsis-associated disseminated intravascular coagulation (DIC) [25]; 50-64.9% (moderate deficiency, 2 points) based on Pereyra et al.'s 61.5% threshold for organ dysfunction [26]; 35-49.9% (severe deficiency, 3 points) aligned with Wada et al.'s definition of severe AT deficiency at <50% [27]; and <35% (critical deficiency, 4 points) (see Appendices).

These align with clinical AT replacement decision points. Consequently, ASOFA scores ranged from 0 to 28 points. K-means clustering was performed using the individual SOFA score components other than Cre, specifically the cardiovascular (mean arterial pressure and inotrope requirement), respiratory (partial pressure of oxygen in arterial blood (PaO₂)/fraction of inspired oxygen (FᵢO₂) ratio), hepatic (total bilirubin), and coagulation (platelet count) components.

Statistical analysis

Continuous variable distribution normality was evaluated using Kolmogorov-Smirnov testing. Categorical data appear as frequencies and percentages. Two-group continuous variable comparisons utilized Mann-Whitney U testing or Student's t-testing, while three-or-more-group comparisons employed ANOVA or Kruskal-Wallis testing. Categorical variable comparisons used chi-square testing, with Fisher's exact testing for expected values below 5. Diagnostic performance was assessed through receiver operating characteristic area under the curve (ROC AUC) analysis. Optimal ROC curve thresholds were established using Youden index calculations.

ASOFA score and AT/Cre ratio prognostic accuracy evaluation employed ROC analysis and K-means clustering. ROC analysis was also performed for the SOFA score, APACHE II score, and the acute DIC score [28]. 

For each identified cluster, patient characteristics and AT/Cre ratio, SOFA score, and ASOFA score prognostic accuracy were analyzed. Model performance was evaluated regarding discharge mortality prediction capability. Prior to clustering, all variables were standardized using a z-score transformation to ensure equal contribution of each SOFA component to the distance calculations. The number of clusters was determined using the Elbow method by assessing the within-cluster sum of squared errors together with clinical interpretability. Spline curves were generated to visualize the relationships between mortality and each severity score.

Continuous variables are presented as medians with interquartile ranges. Statistical significance was defined as p <0.05. All analyses were performed using Python (Python Software Foundation, Wilmington, Delaware, United States).

Multiple imputation using Bayesian methodology addressed missing data. Before imputation, missingness was quantified for all key variables, including demographic, laboratory, and physiological parameters. Age and sex had no missing values (0%). Antithrombin activity and serum creatinine also had no missingness (0%) because AT measurement within 24 hours of ICU admission was an inclusion criterion. Among SOFA-related parameters, PaO₂/FᵢO₂ ratio showed 4.1% missingness, mean arterial pressure 1.1%, inotrope dose 2.6%, platelet count 0.7%, and total bilirubin 3.4%. No variable exceeded 5% missingness. All variables with missing values were included in the Bayesian multiple imputation procedure. No formal sample size calculation was performed; however, the number of outcome events relative to predictors was considered sufficient for exploratory prognostic evaluation.

## Results

**Figure 1:**
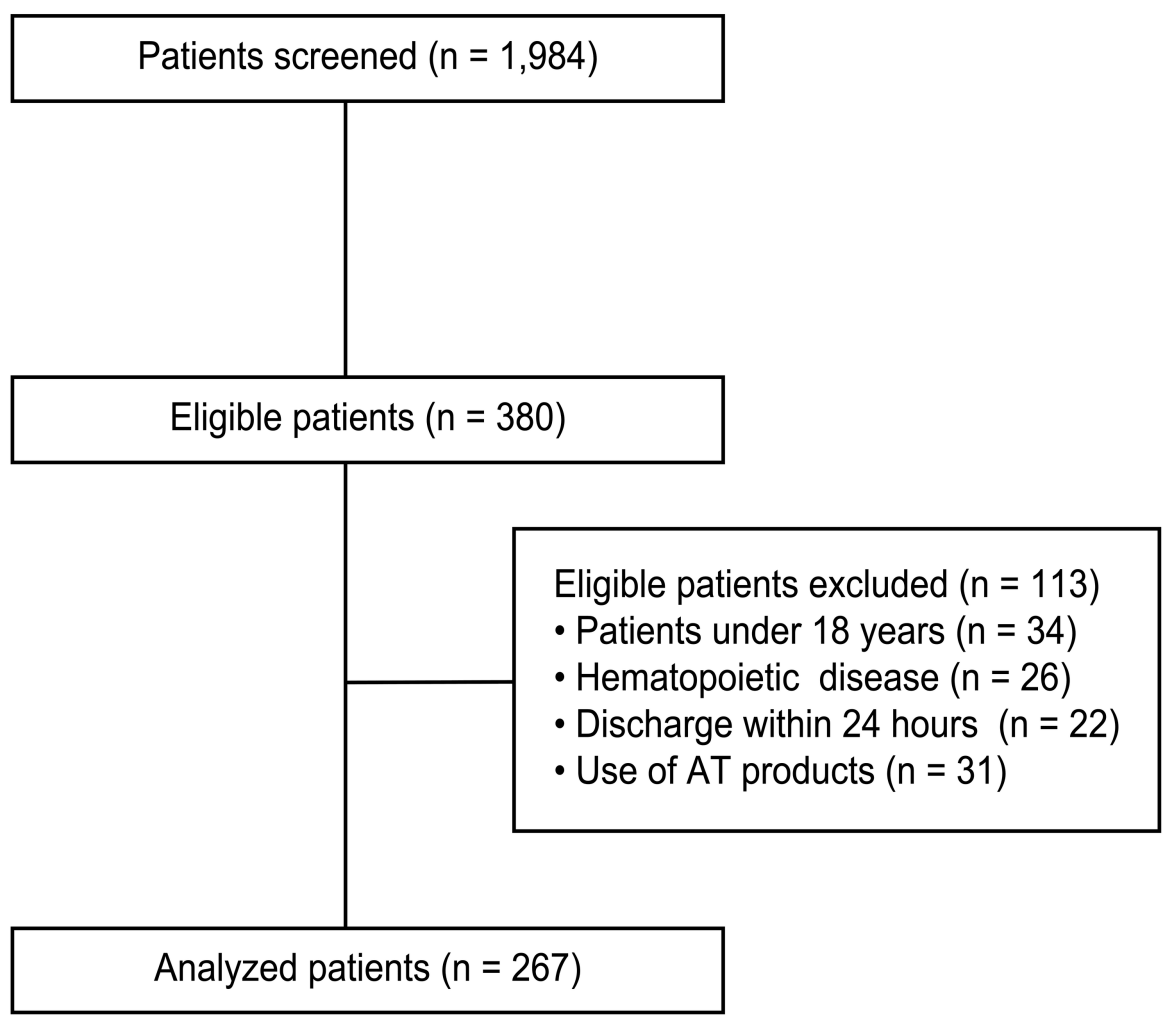
Patient enrollment flow chart AT, antithrombin

During the study period, 1984 patients received ICU admission. Among these, 267 patients met the inclusion criteria, focusing on those with measured AT activity levels meeting study requirements (Figure 1). Most patients were male (60.4%), with a median age of 69 (interquartile range (IQR), 58-77) years. Admission reasons included postoperative cases (58.1%) and emergency admissions (78.3%) (Table 1).

**Table 1:** Characteristics of the study population Data are shown as count (%) for categorical variables and as median (IQR) or mean±SD for continuous variables, as appropriate. Between-group comparisons were performed using Mann-Whitney U test for non-parametric continuous variables, Student's t-test for normally distributed continuous variables, and chi-square test for categorical variables. For Mann-Whitney U tests, the U statistic is reported; for Student's t-test, the t statistic is reported; for chi-square tests, the χ² statistic is reported. Two-sided p<0.05 was considered statistically significant, and significant results are marked with *. Alb, albumin; APACHE, Acute Physiology and Chronic Health Evaluation; AT, antithrombin activity; DIC, aisseminated intravascular coagulation; FDP, fibrin/fibrinogen degradation products; FiO₂, fraction of inspired oxygen; Ht, hematocrit; IQR, interquartile range; PaO₂, partial pressure of arterial oxygen; Plt, platelet count; PT-INR, prothrombin time–international normalized ratio; SD, standard deviation; SOFA, Sequential Organ Failure Assessment; WBC, white blood cell count

Variable	All patients (n=267)	Survivors (n=209)	Non-survivors (n=58)	P value	Stat
Age (years), median (IQR)	69 (58-77)	69 (57-77)	71 (62-79)	0.044 *	U=5.02e+03
Sex (male), n (%)	173 (64.8%)	137 (65.6%)	36 (62.1%)	0.64	χ² = 0.218
Surgery, n (%)	155 (58.1%)	126 (60.3%)	25 (43.1%)	0.011 *	χ² = 4.73
Urgent admission, n (%)	209 (78.3%)	163 (78.0%)	46 (79.3%)	0.471	χ² = 0.041
Ventilation, n (%)	191 (71.5%)	146 (69.9%)	45 (77.6%)	0.188	χ² = 1.113
Inotrope, n (%)	131 (49.1%)	92 (44.0%)	39 (67.2%)	< 0.001 *	χ² = 8.80
WBC (×10^3^/μl), median (IQR)	9.9 (7.1-13.8)	10.3 (7.5-14.0)	8.7 (5.5-11.7)	0.0165 *	U=7.31e+03
Ht (%), median (IQR)	31.4 (27.4-35.7)	31.7 (27.7-35.8)	30.3 (26.9-34.6)	0.21	U=6.71e+03
Plt (×10^4^/μl), median (IQR)	14.0 (9.2-19.1)	15.3 (9.4-19.4)	11.5 (8.6-16.4)	0.0205 *	U=7.27e+03
Alb (g/dl), mean±SD	2.8±0.6	2.8±0.6	2.5±0.7	0.00135 *	t= 3.05
PT-INR, median (IQR)	1.16 (1.05-1.36)	1.13 (1.04-1.31)	1.33 (1.10-1.56)	< 0.001*	U=3.91e+03
AT (%), median (IQR)	66 (53-84.3)	69 (56-86)	56 (44-73)	< 0.001 *	U=7.94e+03
AT/Creatinine (%/mg/dl), median (IQR)	65 (33-98)	72 (42-109)	33 (24-59)	< 0.001 *	U=8.59e+03
FDP (μg/ml), median (IQR)	12.6 (5.8-24.5)	10.1 (5.6-21.7)	16.5 (9.1-91.3)	< 0.001 *	U=3.28e+03
Creatinine (mg/dl), median (IQR)	0.97 (0.70-1.75)	0.93 (0.66-1.40)	1.47 (0.88-2.47)	< 0.001 *	U=3.88e+03
PaO_2_/F_I_O_2,_, median (IQR)	279 (186-369)	288 (206-364)	244 (162-295)	0.287	U=6.62e+03
APACHE Ⅱ score, median (IQR)	17 (12-21)	16 (12-20)	21 (15-25)	< 0.001 *	U=3.67e+03
SOFA score, median (IQR)	7 (4-9)	6 (4-8)	9 (7-11)	< 0.001 *	U=3.11e+03
ASOFA score, median (IQR)	8 (6-10)	7 (5-10)	11 (9-12)	< 0.001 *	U=2.95e+03
Acute DIC score, median (IQR)	2 (1-4)	2 (1-3)	3 (2-4)	< 0.001 *	U=4.18e+03
Predictive mortality (%), median (IQR)	26.2 (16.5-38.9)	23.5 (16.5-35.5)	38.9 (22.9-50.6)	< 0.001 *	U=3.66e+03

**Figure 2:**
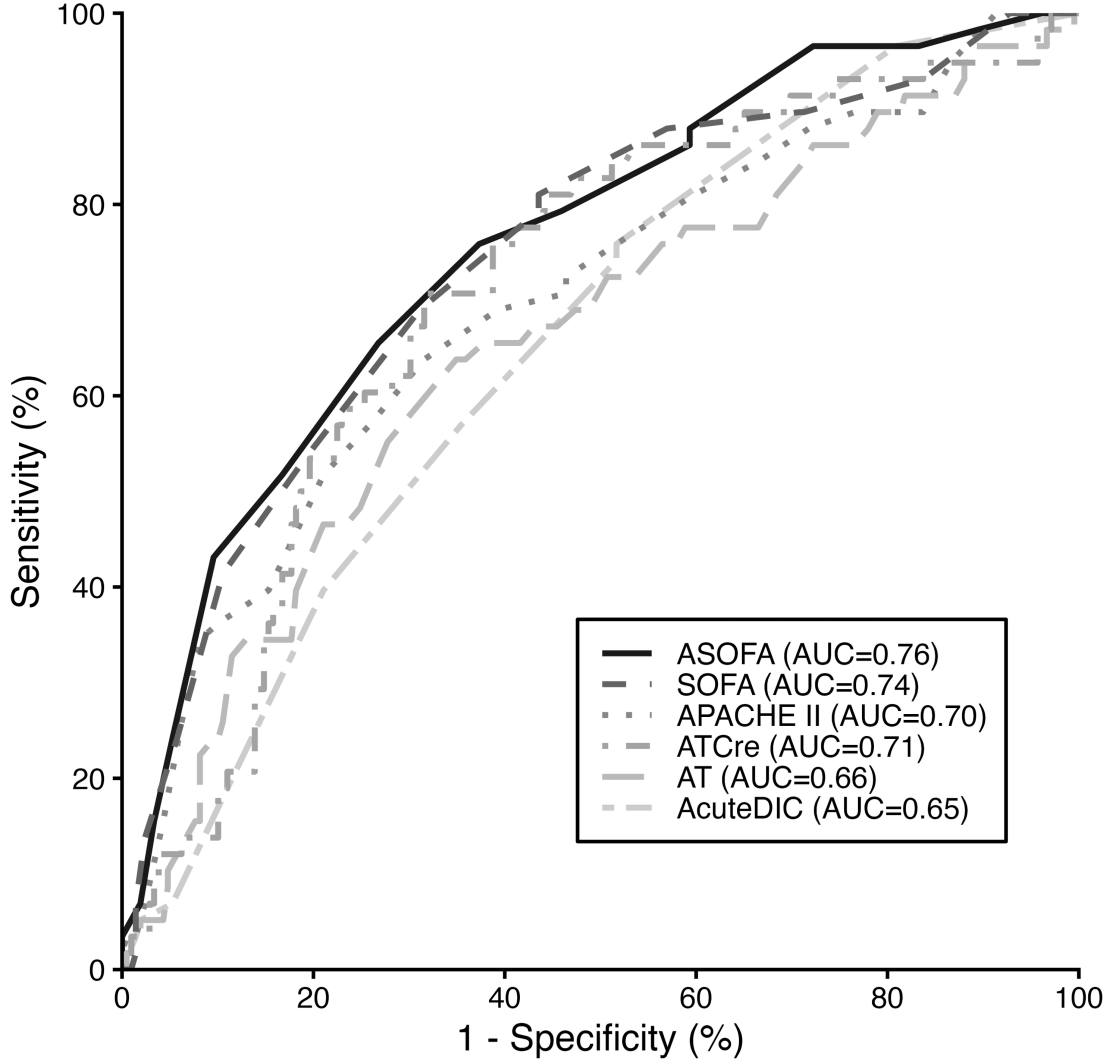
ROC curves for severity scores ROC analysis compares the prognostic performance of six parameters for in-hospital mortality prediction: SOFA (AUC = 0.74), ASOFA (AUC = 0.76), APACHE II (AUC = 0.70), Acute DIC score (AUC = 0.65), antithrombin activity (AT; AUC = 0.66), and the antithrombin/creatinine ratio (AT/Cre; AUC = 0.71). The dashed grey line represents the reference line of no discrimination (AUC = 0.50). APACHE II, Acute Physiology and Chronic Health Evaluation II; ASOFA, Antithrombin-adjusted Sequential Organ Failure Assessment; AT, antithrombin activity; AT/Cre, antithrombin activity to serum creatinine ratio; DIC, disseminated intravascular coagulation score; SOFA, Sequential Organ Failure Assessment; ROC, receiver operating characteristic

Predominant pathologies were sepsis and post-cardiac surgery conditions. Across the population, the AT/Cre ratio demonstrated prognostic capability with an ROC AUC of 0.71, a sensitivity of 0.72, and a specificity of 0.68 at a threshold of 53.6 (Figure 2). SOFA scoring achieved ROC AUC of 0.75, sensitivity of 0.70, and specificity of 0.69 at a threshold of 8.0. 

In Cox proportional hazards modeling, the AT/Cre ratio showed a significant mortality risk association in both univariate and multivariate analyses. In univariate Cox regression analysis, the AT/Cre ratio was associated with reduced mortality risk (hazard ratio (HR) 0.98, 95% confidence interval (CI) 0.98-0.99; Wald χ² =8.1, p <0.005), with a concordance index of 0.70. Following adjustment for predefined confounders in multivariate Cox regression, the AT/Cre ratio remained statistically significant (HR 0.98, 95% CI 0.98-0.99; Wald χ² =7.6, p <0.005), with slightly improved concordance (0.71)

**Figure 3:**
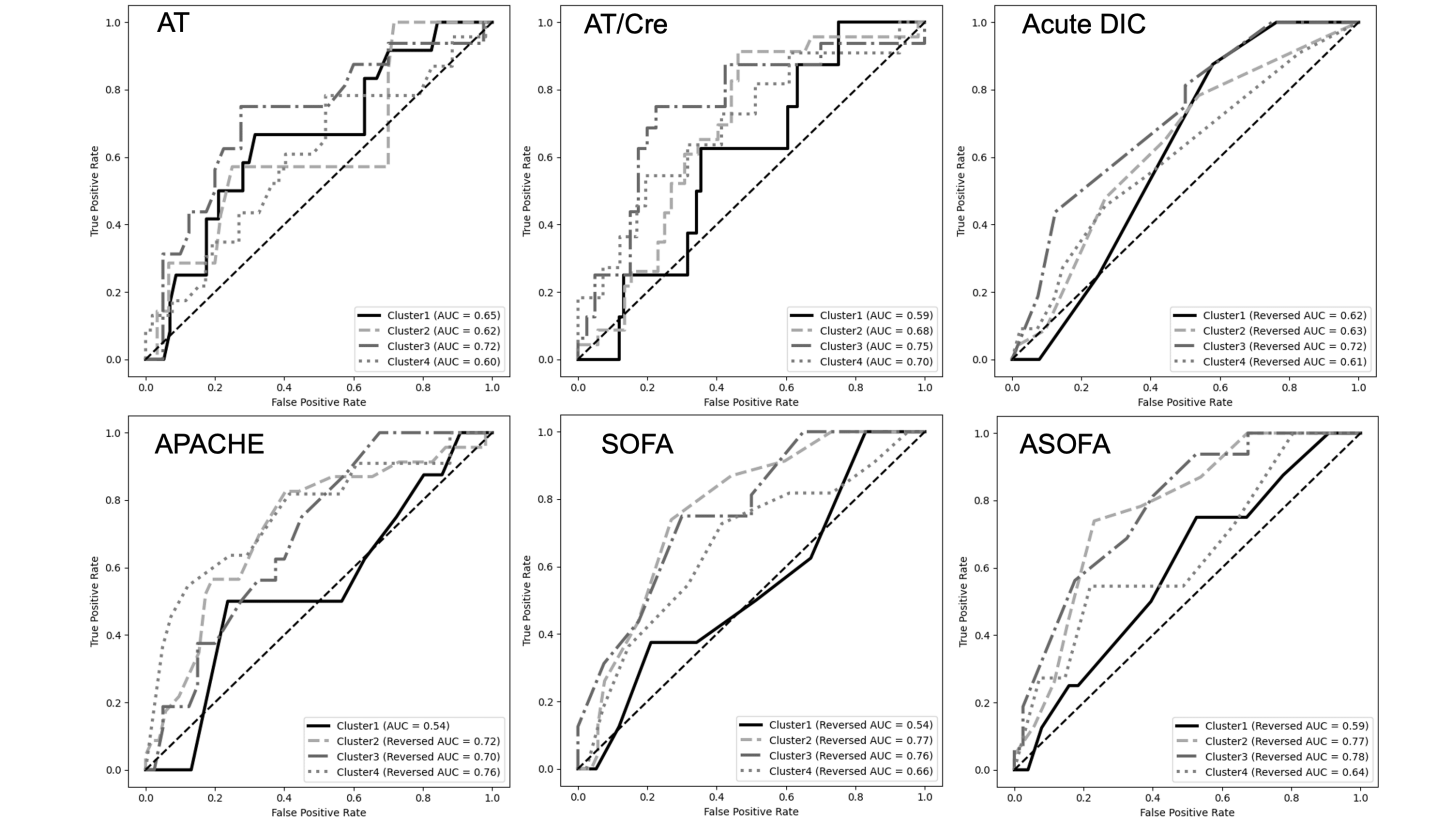
ROC curves comparing model discrimination across clusters ROC analysis was performed to evaluate and compare the discriminative performance of each prognostic model for in-hospital mortality within patient clusters identified by K-means clustering based on SOFA score components, excluding serum creatinine. ROC curves depict the relationship between sensitivity and 1 − specificity for AT, AT/Cre, acute DIC score, APACHE II score, SOFA score, and ASOFA score. AUC values were calculated separately for each model within each cluster to assess cluster-specific predictive performance. In Cluster 3, ASOFA demonstrated the highest discriminative performance for mortality prediction (AUC = 0.78, 95% CI 0.73–0.88). APACHE II, Acute Physiology and Chronic Health Evaluation II; ASOFA, antithrombin-modified Sequential Organ Failure Assessment score; AT, antithrombin activity; AT/Cre, antithrombin activity to serum creatinine ratio; AUC, area under the curve; DIC, disseminated intravascular coagulation (acute DIC score defined by the Japanese Association for Acute Medicine); ROC, receiver operating characteristic; SOFA, Sequential Organ Failure Assessment score

Patient categorization into four clusters using SOFA score parameters through K-means clustering revealed differing AT/Cre values, with each cluster exhibiting distinct prognoses. The population was segregated into four distinctive clusters with unique characteristics (Table 2). AT/Cre ratio was significantly elevated in clusters 1 and 2, indicating more favorable prognoses (AT/Cre ratios for clusters 1, 2, 3, and 4 were 72 (50-123), 76 (44-118), 58 (28-81), and 44 (23-75), respectively (Kruskal-Wallis test, H =24.9, p <0.0001). Post hoc pairwise comparisons with Bonferroni correction demonstrated significantly higher AT/Cre ratios in clusters 1 and 2 compared with clusters 3 and 4 (adjusted p <0.05). Clusters 1 and 2 were characterized by notably low circulatory parameters within the SOFA scoring. In Cluster 2, all SOFA parameters were ≤1 point. Within Cluster 2, neither AT/Cre ratio nor SOFA score demonstrated accuracy (ROC AUC: AT/Cre 0.49, SOFA score 0.49). Cluster 2 also exhibited reduced organ damage across all parameters (Figure 3).

**Table 2:** Comparison of baseline characteristics, laboratory data, severity scores, and outcomes across four patient clusters. Data are presented as median (IQR) for continuous variables, or as percentage (count/total count) for categorical variables. Comparisons across the four clusters were performed using the Kruskal–Wallis test for continuous variables and the chi-square test for categorical variables. For Kruskal–Wallis tests, the H statistic is reported; for chi-square tests, the χ² statistic is reported. When the overall test showed significance (p < 0.05), pairwise comparisons were performed using Dunn's multiple comparison test for Kruskal-Wallis tests. Significance thresholds were set at p < 0.05. *: Indicates a statistically significant difference (p < 0.05) across the clusters. APACHE II, Acute Physiology and Chronic Health Evaluation II; ASOFA, antithrombin-adjusted Sequential Organ Failure Assessment score; AT, antithrombin activity; AT/Cre, antithrombin activity/creatinine ratio; BNP, brain natriuretic peptide; CRP, C-reactive protein; DIC, disseminated intravascular coagulation (referring to the acute DIC score by the Japanese Association for Acute Medicine); FDP, fibrin/fibrinogen degradation products; FiO₂, fraction of inspired oxygen; HCO₃⁻, bicarbonate; IQR, interquartile range; K, potassium; Lac, lactate; Na, sodium; P/F, ratio of partial pressure of arterial oxygen (PaO₂) to fraction of inspired oxygen (FiO₂); PaO₂, partial pressure of arterial oxygen; PTINR, prothrombin time–international normalized ratio; SOFA, Sequential Organ Failure Assessment score

	Cluster 1 (n=84)	Cluster 2 (n=75)	Cluster 3 (n=56)	Cluster 4 (n=52)	P value	Stat
Age (year), median (IQR)	66 (51-77)	70 (60-77)	70 (56–78)	70 (63–75)	0.16	H=2.16
Admission type (Operation), n (%)	60.7 (51/84)	57.3 (43/75)	62.5 (35/56)	50.0 (26/52)	0.38	χ²=2.1
Height (cm), median (IQR)	162 (155-169)	162 (154–169)	162 (157–168)	164 (157–168)	0.89	H=0.45
Weight (kg), median (IQR)	57.8 (49.6-64.7)	60.8 (53.4–69.9)	57.7 (48.0–62.6)	60.1 (51.2–66.9)	0.036*	H=5.66
Gender (male), n (%)	59.5 (50/84)	65.3 (49/75)	64.3 (36/56)	73.1 (38/52)	0.99	χ²=0.033
Body Temperature (℃), median (IQR)	37.0 (36.3-37.5)	36.8 (36.0–37.6)	36.5 (35.8–37.2)	37.1 (36.5–37.8)	0.0044*	H=11
Heart rate (beat/minute), median (IQR)	84 (80-100)	90 (80–105)	90 (80–105)	90 (83–105)	0.25	H=5.5
Mean blood pressure (mmHg), median (IQR)	82 (75-99)	72 (64–81)	75 (65–82)	80.0 (73–100)	< 0.001*	H=28.8
White blood cell count (/μL), median (IQR)	10575 (7430-13998)	9470 (7145–13735)	10425 (7697.5–13325)	9775 (7097.5–14290)	0.98	H=1.24
Platelet count (×10⁴/μL), median (IQR)	16.6 (12.3-20.4)	10.6 (7.8–17.8)	13.6 (8.8–18.8)	15.6 (9.7–19.4)	0.0033*	H=17.1
Hematocrit (%), median (IQR)	31.0 (27.3-35.8)	31.4 (27.8–35.0)	31.5 (29.0–34.2)	31.6 (26.8–35.8)	0.63	H=0.465
Na (mEq/L), median (IQR)	140 (137-143)	142 (138–146)	141 (138–144)	140 (137–143)	0.14	H=4.39
K (mEq/L), median (IQR)	4.0 (3.7-4.4)	4.1 (3.8–4.7)	4.2 (4.0–4.5)	3.9 (3.6–4.2)	0.019*	H=10.1
Albumin (g/dL), median (IQR)	2.7 (2.4-3.1)	2.8 (2.4–3.2)	2.8 (2.2–3.2)	2.8 (2.2–3)	0.73	H=2.14
Creatinine (mg/dL), median (IQR)	0.83 (0.63-1.07)	1.4 (0.9–2.2)	1.1 (0.8–1.8)	0.9 (0.7–1.3)	0.022*	H=20.7
Total Bilirubin (mg/dL), median (IQR)	0.7 (0.5-1.1)	1.0 (0.6–2.0)	0.9 (0.5–1.6)	0.9 (0.5–1.3)	0.016*	H=10.5
CRP (mg/dL), median (IQR)	6.4 (2.7-13.6)	4.3 (1.2–12.0)	3.2 (1.8–11.2)	6.7 (2.2–15.4)	0.33	H=3.68
Antithrombin (%), median (IQR)	71.0 (58.8-86.0)	64.0 (47.5–84.5)	58.0 (50.8–71.8)	65.5 (50.5–87.0)	0.11	H=9.62
AT/Cre (%/(mg/dL)), median (IQR)	83.3 (50.5-127.2)	44.4 (23.6–75)	58.8 (29.0–81.3)	70.1 (45.7–95.9)	0.015*	H=24.6
BNP (pg/mL), median (IQR)	717 (306-754)	726 (258–770)	702 (179–748)	656 (75–754)	0.51	H=4.07
PTINR, median (IQR)	1.21 (1.09-1.32)	1.1 (1–1.5)	1.2 (1.0–1.5)	1.2 (1.1–1.3)	0.69	H=1.9
Fibrinogen (mg/dL), median (IQR)	355 (251-449)	307 (234–383)	323 (228–400)	351 (257–519)	0.032*	H=5.71
FDP (μg/mL), median (IQR)	15.9 (7.2-27.5)	13.1 (6.0–33.8)	12.9 (5.6–24.7)	12.8 (6.2–24.5)	0.67	H=1.86
pH, median (IQR)	7.39 (7.35-7.44)	7.40 (7.30–7.40)	7.40 (7.30–7.40)	7.40 (7.30–7.40)	0.54	H=1.27
P/F, median (IQR)	349 (313-406)	188 (132–247)	396 (328–475)	170 (135–235)	< 0.001*	H=185
HCO_3-_ (mEq/L), median (IQR)	22.8 (20.8-24.7)	23.5 (20.2–26.4)	22.2 (19.2–25.6)	24.3 (22.4–26.8)	0.11	H=6.01
Lac (mmol/L), median (IQR)	3.0 (1.8-4.9)	3.0 (1.8–4.9)	2.7 (1.8–4.2)	3.2 (2.1–4.2)	0.75	H=13.1
APACHE II score, median (IQR)	15.0 (10.0-18.0)	20 (15–24)	15 (12–19.2)	18.5 (14.8–21)	< 0.001*	H=33.6
SOFA score, median (IQR)	5.0 (3.0-6.0)	9 (7–10.5)	7 (5.8–8.2)	6 (4.8–8)	< 0.001*	H=80.2
Circulation, median (IQR)	0.0 (0.0-0.0)	2.0 (2.0,3.0)	2.0 (2.0,3.0)	0.0 (0.0,0.0)	< 0.001*	H=227
Kidney, median (IQR)	0.0 (0.0-0.0)	1.0 (0.0,2.0)	0.0 (0.0,1.0)	0.0 (0.0,1.0)	< 0.001*	H=21.7
Liver, median (IQR)	0.0 (0.0-0.25)	0.0 (0.0,2.0)	0.0 (0.0,1.0)	0.0 (0.0,1.0)	< 0.001*	H=11.2
Respiration, median (IQR)	1.0 (0.0-1.0)	3.0 (2.0,3.0)	0.5 (0.0,1.0)	3.0 (2.0,3.0)	< 0.001*	H=195
Coagulation, median (IQR)	1.0 (1.0-1.0)	1.0 (1.0,2.0)	1.0 (1.0,2.0)	1.0 (1.0,2.0)	< 0.001*	H=14.7
Consciousness, median (IQR)	1.0 (1.0-1.0)	2.0 (1.0,2.0)	1.0 (1.0,2.0)	2.0 (1.0,2.0)	< 0.001*	H=58.1
ASOFA score, median (IQR)	6 (4–7)	10 (9–12)	9 (6.9–11)	7.5 (6–10)	< 0.001*	H=76.8
Acute DIC score, median (IQR)	2.0 (1.0-3.3)	3 (1–4)	2 (1–3)	1 (1–3)	0.11	H=6.06
Predicted mortality (%), median (IQR)	21.0 (11.3-29.1)	35.5 (21.0-49.7)	21.0 (14.6-33.1)	30.7 (20.4-38.9)	< 0.001*	H=33.8
7-day mortality, n (%)	2.4 (2/84)	6.7 (5/75)	12.5 (7/56)	3.8 (2/52)	0.084	χ²=6.64
28-day mortality, n (%)	6.0 (5/84)	16.0 (12/75)	20.0 (11/56)	15.4 (8/52)	0.090	χ²=6.47
Admission mortality, n (%)	9.5 (8/84)	30.7 (23/75)	28.6 (16/56)	21.2 (11/52)	0.012*	χ²=12.4

**Figure 4:**
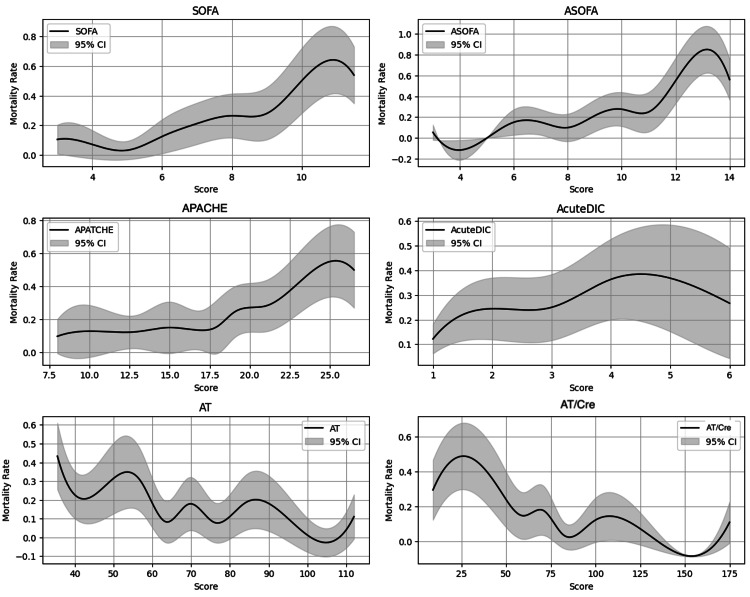
Spline smoothed mortality curves with 95% confidence Intervals This figure illustrates the association between severity scores/biomarkers and predicted mortality risk using spline-smoothed regression models. The solid black lines represent the spline-smoothed mortality curves, while the gray shaded areas indicate the 95% confidence intervals. Mortality risk is shown as a function of SOFA, ASOFA, APACHE II, acute DIC score, AT, and AT/Cre. Spline curves were used for visualization of non-linear relationships and were not intended for formal hypothesis testing. APACHE II, Acute Physiology and Chronic Health Evaluation II; ASOFA, antithrombin-adjusted Sequential Organ Failure Assessment score; AT, antithrombin activity; AT/Cre, antithrombin activity/creatinine ratio; DIC, disseminated intravascular coagulation (referring to the acute DIC score by the Japanese Association for Acute Medicine); SOFA, Sequential Organ Failure Assessment score

Spline curve distribution illustrated mortality-predictive accuracy relationships (Figure 4). Elevated SOFA and APACHE II scores correlated with poor prognoses, while decreased AT alone and AT/Cre values correlated with poor prognoses. Notably, AT/Cre ratio showed strong association with poor prognosis at lower values.

## Discussion

This investigation evaluated AT/Cre ratio prognostic capabilities in critically ill patients, addressing the need for streamlined prognostic approaches utilizing limited parameter sets as emphasized in current critical care literature [3-8]. The AT/Cre ratio demonstrated prognostic ability with an AUC of 0.72, which was close to but slightly lower than the AUC of 0.75 observed for the SOFA score. Thus, while the ratio shows clinically relevant discrimination, it does not exceed the performance of existing multivariable tools. Our findings align with previous investigations demonstrating AT depletion's prognostic significance in severe infections and multi-organ failure [13-18]. 

The AT/Cre ratio's predictive capability supports the hypothesis that consolidating coagulation disturbances and renal dysfunction into a unified metric provides enhanced assessment of their interaction, as suggested by preliminary investigations [21]. However, based on the present results, the ratio should be interpreted as offering performance comparable to SOFA rather than superior prognostic ability. Importantly, our study did not test whether AT/Cre confers incremental prognostic value beyond SOFA (e.g., through combined modeling), and, therefore, we refrain from claiming additive benefit.

Additionally, ASOFA scoring demonstrated significant predictive accuracy (comparable to traditional SOFA), validating the concept of incorporating AT activity into established scoring systems to better reflect endothelial dysfunction and coagulation abnormalities [19]. Nevertheless, similar to the AT/Cre ratio, the current data do not establish superiority over conventional SOFA scoring.

Cluster analysis yielded further insights into the conditions under which the AT/Cre ratio performs well. In clusters with greater organ dysfunction, the ratio showed clear prognostic separation, whereas in Cluster 2, characterized by uniformly preserved organ function and minimal physiological derangement, both AT/Cre and SOFA exhibited poor discrimination (AUC 0.49). This likely reflects a ceiling effect in low-risk groups, where limited physiological variability diminishes the ability of any biomarker or score to detect mortality differences. These findings suggest that the AT/Cre ratio reflects pathophysiological characteristics beyond simple numerical values, supporting the established correlation between hemostatic dysfunction and critical illness severity [9-12]. Cox proportional hazards analysis confirmed AT/Cre ratio as an independent prognostic predictor (HR 0.98 [95%CI 0.98-0.99]), consistent with literature demonstrating AT's prognostic value in critically ill patients [13-18]. Although the HR per unit increase was statistically significant, its effect size was small (2% risk reduction per unit increase in the ratio), underscoring that the AT/Cre ratio is more clinically interpretable when used as a categorized or threshold-based marker rather than as a purely continuous predictor. Notably, within Cluster 1 (mild organ dysfunction), both AT/Cre ratio and SOFA score showed limited predictive accuracy (AUC 0.59), suggesting that current prognostic tools may have reduced utility in patients with preserved organ function. However, ASOFA scoring maintained predictive accuracy across different clusters, supporting its robustness compared to traditional approaches.

Limitations of the study

Study limitations include a retrospective design; a major source of selection bias arises from including only patients who had AT activity measured. AT testing is typically ordered for patients in whom coagulopathy is suspected or in those who are more severely ill, which likely resulted in a cohort enriched for higher-acuity patients. This limitation substantially restricts generalizability to unselected ICU populations and must be interpreted as such.

Second, although the study was conducted at two centers, the absence of an external validation cohort increases the risk of overfitting, particularly regarding the observed AUC values. Consequently, the predictive performance reported here should be interpreted as internal estimates rather than generalizable metrics. Future studies should incorporate external and temporal validation to confirm model stability and reproducibility.

Third, AT measurement variability, including the use of two different analyzers and unavoidable differences in sampling timing, may introduce measurement noise that could affect prognostic performance.

Finally, the sample size was limited, particularly within clusters, which restricts subgroup inference. Future prospective studies should investigate temporal AT/Cre trajectory changes, examine performance in disease-specific subgroups, and validate the utility of the AT/Cre ratio and ASOFA scoring systems in broader ICU populations.

## Conclusions

This study demonstrates that the AT/Cre ratio may be a useful prognostic marker in critically ill patients admitted to ICUs. Multivariate analysis confirmed that the AT/Cre ratio functions as an independent prognostic predictor, particularly in patients with combined coagulation disorders and renal dysfunction.

Cluster analysis revealed that AT/Cre ratio predictive accuracy is particularly useful in patients with multiple organ dysfunction, while ASOFA scoring maintained predictive accuracy across different patient clusters. The AT/Cre ratio derives from routinely measured laboratory parameters, making it highly practical for clinical implementation. Clinical application of AT/Cre ratio and ASOFA scoring shows promise for early risk stratification in critically ill patients, warranting further validation through prospective studies.

## Appendices

**Table 3:** Antithrombin-modified SOFA scoring rubric The ASOFA score was developed by modifying the original Sequential Organ Failure Assessment (SOFA) score through the addition of antithrombin (AT) activity as an independent organ dysfunction component. Each organ system—respiratory, coagulation, liver, cardiovascular, central nervous system, renal function, and antithrombin activity—is scored from 0 to 4 according to the severity of dysfunction, yielding a total ASOFA score ranging from 0 to 28 points. Respiratory function is assessed using the PaO₂/FiO₂ ratio with consideration of respiratory support. Coagulation is evaluated based on platelet count, liver function by serum bilirubin concentration, cardiovascular function by mean arterial pressure (MAP) and vasoactive agent requirements, central nervous system function by the Glasgow Coma Scale, and renal function by serum creatinine concentration or urine output. Antithrombin activity (%) is categorized into five severity levels and incorporated as an additional component of the score. The ASOFA score was created by the authors for exploratory prognostic evaluation in critically ill patients. MAP, mean arterial pressure; Nad, norepinephrine; DOB, dobutamine; DOA, dopamine; Ad, epinephrine; γ, µg/kg per minute; Pao2, partial pressure of arterial oxygen; Fio2, fraction of inspired oxygen

Parameters	Score=0	Score=1	Score=2	Score=3	Score=4
Respiratory Pao_2_/Fio_2_ (mmHg)	≥400	<400	<300	<200 + respiratory support.	<100 + respiratory support.
Coagulation Platelets Count (×10^3^/μL)	≥150	<150	<100	<50	<20
Liver Bilirubin (mg/dL)	<1.2	1.2~1.9	2.0~5.9	6.0~11.9	>12.0
Circulation MAP (mmHg)	MAP ≥70	MAP <70	DOA <5γ or DOB (any dose)	DOA 5.1~15 or Ad ≤0.1γ or Nad ≤0.1γ	DOA >15γ or Ad >0.1γ or Nad >0,1γ
Central Nervous System Glasgow Coma Scale	15	13~14	10~12	6~9	<6
Renal Creatinine (mg/dL) or Urine Output (mL/d)	<1.2	1.2~1.9	2.0~3.4	3.5~4.9	≥5.0
Antithrombin Activity (%)	≥80	65~79.9	50~64.9	35~49.9	<35
